# Rembrandt’s Jewish Physician—Dr Ephraim Bueno (1599–1665): A Brief Medical History

**DOI:** 10.5041/RMMJ.10110

**Published:** 2013-04-30

**Authors:** George M. Weisz, William R. Albury

**Affiliations:** School of Humanities, University of New England, Armidale, New South Wales, Australia

**Keywords:** Dr Bueno, painting, physician, Rembrandt

## Abstract

Medicine in the Middle Ages was, and ever since remained, one of the main preoccupations of the professionally restricted Jews. One of the medical dynasties on the Iberian Peninsula was the Bueno (Bonus) family. Following the expulsion of the Jews from Spain and their spread in Europe, these Iberian physicians became successful everywhere—just as the Buenos were in the Netherlands.

The contribution to the Netherlands by the exiled Jewish community of Iberian origin is well known and was indeed a significant one in the Golden Age of the seventeenth century.

It was in this age of reason that an intellectual center evolved in Amsterdam’s *Judebeestraat* (Jewish street) in the Jewish Quarter (not ghetto), neighboring the beautiful Portuguese synagogue. It was a center which fostered the development of the arts (by the painter Rembrandt Harmenszoon van Rijn), of Jewish scholarship (by Manasseh ben Israel, a Torah teacher and publisher of religious books), and of medicine (by members of the Bueno medical dynasty).

Rembrandt (1606–1669), the great artist, had settled in the Jewish quarter with his family—his wife and two daughters, and later on his second wife and a son. They lived fairly long lives, requiring frequent medical assistance for births, children’s diseases, and adult illnesses.

Rembrandt’s interest in Biblical stories is well represented in his many paintings based on the Old Testament (*Jewish Wedding*, *A Young Jew*, *The Saving of Moses*, etc.).^1^ The painting of *Bathsheba with David’s Letter*^2^ depicts a retracted skin deformity on the model’s exposed left breast, a sign of mastitis or perhaps a scirrhous type of carcinoma.^3^

Toward the end of his life, after his entire family had perished, Rembrandt was unable to satisfy his debtors and was evicted from his home. He died in poverty and was buried in a common grave in Westerkerk cemetery in Amsterdam.^4^,^5^

The second figure in the intellectual center which flourished in the Jewish quarter of Amsterdam was Rabbi Manasseh ben Israel (1604–1657).

Born as Manuel Diaz Soeiro in Portugal, he was brought to Amsterdam as a young child. He became a brilliant Jewish theologian, wrote religious texts in five languages, and in 1626 founded the first Hebrew printing press in the Netherlands. His image is known to us from the portraits by Rembrandt and others.^6^

Ben Israel published on religious topics and engaged in diplomatic and scholarly exchanges with leading Puritan theologians and government officials in England. He was tireless in seeking to obtain permission for Jews to be readmitted in England, from which country they had been banished since 1290. He obtained an unofficial permit from Oliver Cromwell in 1656, and after his death a charter was granted by Charles II in 1664.

His most famous book, *El Conciliador* (1632–1651), was intended to make the Old Testament more accessible to simple people and Judaism more understandable to the Gentiles. This work made him known to both Jewish and Christian scholars throughout Western Europe.

The third participant in the intellectual center of Amsterdam’s Jewish quarter was Dr Ephraim Bueno, alias Martin Alvarez. Who was this physician?

The Bueno medical dynasty flourished in the Netherlands after having been thrice exiled from other countries. At first, being exiled from their birthplace in Spain, the Buenos settled in Portugal. The Jews remained in their new country until 1498. After their fortunes had been exhausted, the king expelled them unless they converted, which instantly exposed them to the Inquisition. Once they left, they needed an alias name. In order to protect the Bueno family members left behind, Ephraim became Martin Alvarez.

The Buenos then settled in southern France where, unlike in Spain, they were accepted after conversion and were not persecuted for clandestinely practicing their old religion.

At that time Jews were permitted to study medicine in France, but not to practice the profession. This situation continued until 1615, when once again they were exiled. Their next refuge was in the semi-tolerant Dutch lands.

The Bueno family members listed in the biographical dictionary of Dutch physicians are:

- Abraham, practiced medicine until 1633;
- Benjamin, eventually emigrated to New York and died in 1683;
- Jacob, a graduate of Salamanca Medical School, practiced in Amsterdam until 1661;
- Joseph Morenu, practiced in Amsterdam until 1669;
- Solomon, practiced in Amsterdam until 1681;
- Joseph, a graduate of Bordeaux, served as a private physician to the Regent of the Netherlands until 1631; and his son
- Ephraim, born in 1599 in the village Castello Rodrigo in Portugal, graduate of Bordeaux in 1641, practiced medicine in Amsterdam until 1665.^7^,^8^

The tolerance of the Dutch was well known, but it was incomplete. Although Jews with foreign degrees were permitted to engage in medicine as general practitioners, tolerance was not extended to tertiary education. To obtain a higher degree, several members of the dynasty, like Ephraim, studied in Bordeaux—and did so with distinction—but in Ephraim’s case this was only late in life, namely at the age of 43 (according to the Hebrew calendar).The reason for this delayed graduation is not clear. It is likely that he practiced as a general physician in Amsterdam but needed the doctorate for a higher position.

The Bordeaux University Archives describe in detail the ceremony where Ephraim was awarded his doctorate. The three examiners were all descendants of converted Jews, so-called Marranos, whose families had lived in southern France for generations. The patron of the thesis, Professor Lopes (an old friend of Ephraim’s father), accorded him the title of “Magnus in Medicina.” From there, Ephraim joined the some 400 Portuguese Jews in Amsterdam.

Apart from practicing medicine, he was also a scholar of the Bible. Indeed, together with Ben Manasseh and Jonah Abravanel, Ephraim published poetry, translated into Spanish the *Psalms of David*, and in 1650 published *Pene Rabbah*, an index to the biblical passages found in the *Midrash Rabbah*. He also founded the charitable organization “Torah Or” in Amsterdam. He must have been appreciated as a physician as he attended, together with his father Joseph, the Regent of the Netherlands, the Prince of Orange.

Rembrandt depicted his physician Ephraim on two occasions. It is not known if these portraits were commissioned; possibly they were honoraria for medical services provided by Dr Bueno.

There is firstly an etching of 1647, *Dr Bueno Descending the Stairs*, where he is shown in well-to-do attire with the obvious ring on his index finger representing the insignia of the medical community (Figure 1).^1^ Prints of this etching are found today in museums around the world, such as the Metropolitan Museum in New York and the National Gallery of Victoria in Melbourne, Australia, etc.

**Figure 1.**
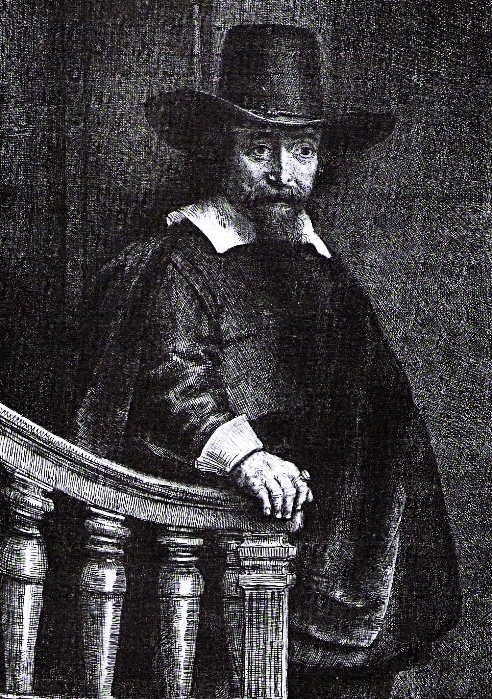
**Rembrandt’s etching of Dr Ephraim Bueno, 1647 (in public domain).**

Rembrandt’s second image of his private physician was an oil portrait, today in the Rijksmuseum, Amsterdam.^9^ This oil painting has an interesting provenance: originally it was in the possession of the Six family and remained so for over two centuries. Then, in the early twentieth century, it was bought by the Jewish banker Fritz Mannheimer. The image of this Jewish physician was then purchased on behalf of Hitler’s intended “Fuehrer Museum” in Linz, Austria (which never eventuated), and it was transferred to the Central Collection Point in Munich in 1942. After the fall of the Third Reich the painting was returned to the Netherlands in 1948 and was finally transferred to the Amsterdam Rijksmuseum in 1960.

In 1656 Ephraim, as an old man, was also etched by another famous artist, Jan Lievens (Figure 2). The figure is seated, wearing a Kippah, and at the bottom of the print is written: “Dr Ephraim Bonus, Medicus Hebraeus ... Magnus in Medicis.”

**Figure 2.**
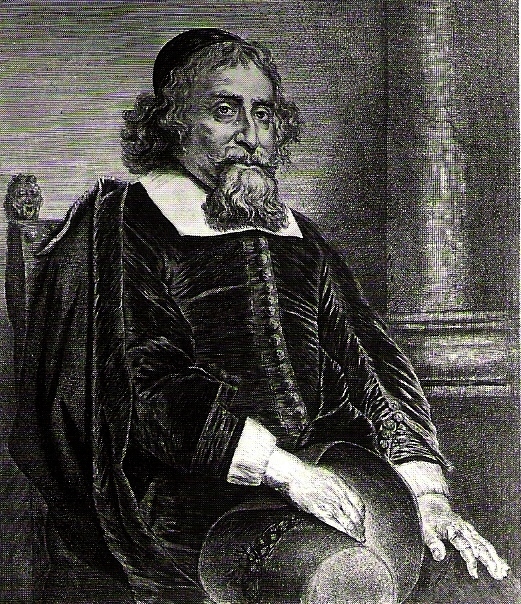
**Lievens’ etching of the elderly Dr Ephraim Bueno (in public domain).**

Ephraim Iskiau Bueno was born Jewish, made a lifetime contribution to medicine, and died as a Jew. His was buried in the Ouderkerk Portuguese Jewish cemetery, Amsterdam. The gravestone gives the date of his death as 30 Hesvan in the year 5426 of the Jewish calendar, which fell on 8 November 1665 (Figure 3).

**Figure 3.**
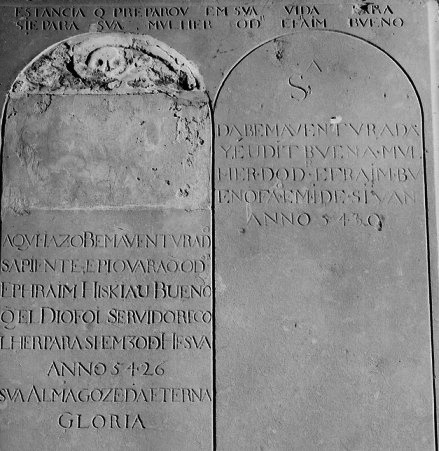
**The grave of Dr Ephraim Bueno.**
